# Anti-obesity effects of heat-transformed green tea extract through the activation of adipose tissue thermogenesis

**DOI:** 10.1186/s12986-022-00648-6

**Published:** 2022-03-03

**Authors:** Hyeonyeong Im, Jaewon Lee, Kyungmin Kim, Yeonho Son, Yun-Hee Lee

**Affiliations:** College of Pharmacy and Research Institute of Pharmaceutical Sciences, Seoul National University, 20-Room # 428,1 Gwanak-ro, Gwanak-gu, Seoul, 08826 Republic of Korea

**Keywords:** HTGT, EMIQ, Adipose tissue, Thermogenic activation, Obesity

## Abstract

**Background:**

Adipose tissue thermogenesis is a potential therapeutic target to increase energy expenditure and thereby combat obesity. The aim of the present study was to investigate the thermogenic and anti-obesity effects of heat-transformed green tea extract (HTGT) and enzymatically modified isoquercetin (EMIQ).

**Methods:**

Immortalized brown pre-adipocytes and C3H10T1/2 cells were used for in vitro analyses. A high-fat diet (HFD)-induced obesity mouse model and CIDEA-reporter mice were used for in vivo experiments. The effects of HTGT and EMIQ on mitochondrial metabolism were evaluated by immunoblot, mitochondrial staining, and oxygen consumption rate analyses. In vivo anti-obesity effects of HTGT and EMIQ were measured using indirect calorimetry, body composition analyses, glucose tolerance tests, and histochemical analyses.

**Results:**

Co-treatment with HTGT and EMIQ (50 μg/mL each) for 48 h increased brown adipocyte marker and mitochondrial protein levels (UCP1 and COXIV) in brown adipocytes by 2.9-fold, while the maximal and basal oxygen consumption rates increased by 1.57- and 1.39-fold, respectively. Consistently, HTGT and EMIQ treatment increased the fluorescence intensity of mitochondrial staining in C3H10T1/2 adipocytes by 1.68-fold. The combination of HTGT and EMIQ (100 mg/kg each) increased the expression levels of brown adipocyte markers and mitochondrial proteins in adipose tissue. Two weeks of HTGT and EMIQ treatment (100 mg/kg each) led to a loss of 3% body weight and 7.09% of body fat. Furthermore, the treatment increased energy expenditure by 8.95% and improved glucose tolerance in HFD-fed mice.

**Conclusions:**

The current study demonstrated that HTGT and EMIQ have in vivo anti-obesity effects partly by increasing mitochondrial metabolism in adipocytes. Our findings suggest that a combination of HTGT and EMIQ is a promising therapeutic agent for the treatment of obesity and related metabolic diseases.

**Supplementary Information:**

The online version contains supplementary material available at 10.1186/s12986-022-00648-6.

## Background

Obesity is characterized by excess accumulation of adipose tissue due to an imbalance between calorie intake and energy expenditure, and is a risk factor for the development of type 2 diabetes and other metabolic diseases [1–3]. The steady increase in obesity and obesity-related metabolic diseases affirms the need for new approaches in the development of anti-obesity therapeutics.

Adipose tissue is categorized as brown adipose tissue (BAT) and white adipose tissue (WAT). Extra energy intake is stored as triglycerides in the lipid droplets of adipocytes, primarily in WAT. WAT mobilizes free fatty acids into the circulation via lipolysis, the process by which triglycerides are hydrolyzed, in response to systemic energy demands [4]. Lipolysis is mainly regulated by the beta-adrenergic receptor and its downstream cAMP-dependent protein kinase A (PKA) signaling pathways. PKA, directly and indirectly, activates lipolytic enzymes, including hormone-sensitive lipase (HSL) and adipose triglyceride lipase (ATGL) [4].

In contrast, BAT is a specialized thermoregulatory organ that dissipates energy as heat via uncoupling protein 1 (UCP1) expression [3]. WAT can recruit brown-like adipocytes (beige adipocytes) and this process is called “browning of WAT” [3, 5]. Thus, the activation and recruitment of BAT metabolism are regarded as a therapeutic target to increase energy expenditure and thereby combat obesity. Browning of WAT can be induced by cold exposure and activation of the beta-adrenergic signaling pathway [5]. In addition to these canonical activators of thermogenesis, several phytochemicals, including components in green tea extract (GTE) and enzymatically modified isoquercitrin (EMIQ), have the potential to induce UCP1 expression and thermogenic programs in adipose tissue [6].

Clinical and preclinical studies support the anti-obesity effects of GTE [7–9]; it reduced the body weight and body mass index of obese individuals in clinical trials [10–12]. Heating processes can modify the chemical composition of the GTE [13, 14]. Several studies have demonstrated that heating increases the concentration of catechins, such as gallocatechin and gallocatechin gallate, while it lowers epicatechin levels such as EGCG. Rats fed a diet containing heat-treated tea catechins showed decreased visceral fat, hepatic triacylglycerol, serum triacylglycerol, and fatty acid synthase activity, suggesting its anti-obesity effect [15]. However, the effects of heat-modified green tea extract on adipose tissue thermogenesis or browning of WAT have not been investigated.

In this study, we investigated the synergistic anti-obesity effects of a combination of HTGT and EMIQ using in vitro adipocytes and HFD-induced obesity mouse model.

## Methods and materials

### Materials

Heat transformed green tea (HTGT: Lot #. 180426) was produced as described previously [16]. Briefly, dried leaf of green tea was extracted with 50% ethanol and heated at 115 °C for 5 h to facilitate the conversion of epigallocatechin gallate to gallocatechin gallate (GCG). Enzymatically-modified isoquercitrin (EMIQ: Lot #. 20191102) were prepared by enzymatic hydrolysis of rutin, followed by transglycosylation reaction [17]. HTGT and EMIQ were provided by Amorepacific Corp. Gallocatechin gallate (GCG) and mirabegron were purchased from Sigma (USA).

### Cell culture

Brown adipocyte cell line was established by immortalization of preadipocytes obtained from interscapular BAT of mice as previously described [18]. Immortalized preadipocytes were cultured in growth medium (Dulbecco’s modified Eagle’s medium (Welgene, South Korea) supplemented with 10% fetal bovine serum (Gibco, USA) and 1% penicillin/streptomycin (Thermo Fisher, USA)) at 37 °C in a humidified atmosphere with 5% CO_2_. For adipocyte differentiation, confluent preadipocytes were exposed to differentiation medium containing 2.5 mM of isobutylmethylxanthine (IBMX, Cayman, USA), 1 μM of dexamethasone (Cayman, USA), 10 μg/mL of insulin (Sigma, USA), 125 μM of indomethacin (Cayman, USA) and 1 nM of triiodothyronine (T3, Cayman, USA) for 3 days followed by maintenance medium containing insulin (10 μg/mL) and T3 (1 nM) for 3 days.

C3H10T1/2 (ATCC, USA) cells were cultured in growth medium. For adipogenic differentiation, C3H10T1/2 cells were exposed to DMEM containing 20 ng/mL of bone morphogenetic protein 4 (BMP4, R&D systems, USA) for 2 days, differentiated in differentiation medium for 3 days, and exposed to maintenance medium for 3 days.

### Animals

6-week-old male C57BL/6 mice (JA Bio, South Korea) and Cell death-inducing DNA fragmentation factor-like effector A (CIDEA) reporter mice (Cidea^tm1(Luc2/tdTomato)Yjl^/KMPC) were used for in vivo experiments following approved protocols by Institutional Animal Care and Use Committees of Seoul National University (SNU-200904-7-3, SNU-201221-3). CIDEA reporter mice were obtained as described previously [19]. All experiments were performed at Animal Center for Pharmaceutical Research in Seoul National University, where mice were housed at 22 ± 1 °C, 12-h light/12-h dark cycle condition with free access to food and water.

Mice were divided into the following three groups: a mirabegron (Mira) group, a co-treatment of HTGT and EMIQ group (H + E) and a vehicle group (CTL). Mirabegron (10 mg/kg) was treated intraperitoneally once daily for 2 weeks as previously described with minor modification [20]. Combination of HTGT and EMIQ was dissolved in distilled water and treated orally at high dose (100 + 100 mg/kg) or low dose (50 + 50 mg/kg) once daily for 2 weeks. Control groups were received vehicle orally once daily for 2 weeks.

For diet-induced obesity mouse model, mice were fed with a 60% fat diet (D12492, Research Diets, USA) for 8 weeks. Then mice were intraperitoneally administered with mirabegron (10 mg/kg) or orally treated with combination of HTGT and EMIQ (100 + 100 mg/kg) once daily for 2 weeks. Vehicle treated control group was included.

### Immunoblot analysis

Immunoblot analysis was performed as previously described [21]. For the protein extraction, cells were lysed using RIPA Lysis and Extraction Buffer (Thermo Fisher, USA) containing SIGMAFAST Protease Inhibitor (Sigma, USA) and PhosSTOP phosphatase inhibitor (Roche, Switzerland). Adipose tissues were lysed using PRO-PREP Protein Extraction Solution (iNtRON Biotechnology, South Korea) containing SIGMAFAST Protease Inhibitor and PhosSTOP phosphatase inhibitor. Protein concentration was measured using Pierce BCA Protein Assay Kit (Thermo Fisher, USA) and MultiSkan GO spectrophotometry (Thermo Fisher, USA) at 562 nm. Proteins were separated on 12% SDS–PAGE gel and subsequently transferred to Immun-Blot PVDF membrane (Bio-Rad, USA). Membranes were placed in blocking buffer (5% bovine serum albumin or 5% powdered skim milk in TBST) for 1 h at room temperature and then incubated with primary antibody at 4 °C overnight. Subsequently, membranes were washed and incubated with secondary antibody for 1 h at room temperature. The primary antibodies are listed in Additional file 1: Table S1. Horseradish peroxidase-conjugated secondary anti-mouse and anti-rabbit (Thermo Fisher, USA) were used. Blots were detected with Fusion Solo chemiluminescence imaging system (Vilber Lourmat, France) and analyzed with EvolutionCapt software (version 17.03). Intensities of band were quantified with National Institutes of Health Image J software (version 1.52a).

### Fluorescence staining of mitochondria and neutral lipid

Cells were incubated with MitoTracker Red CMXRos (1:3000; Thermo Fisher, USA) [22] and BODIPY™ 493/503 (4,4-Difluoro-1,3,5,7,8-Pentamethyl-4-Bora-3a,4a-Diaza-s-Indacene; 1:5000; Thermo Fisher, USA) [23] diluted in growth medium for 15 min at 37 °C for mitochondria and neutral lipid staining. Images were acquired by LSM800 confocal microscope (Zeiss, Germany) and analyzed with Zen software (version 3.0).

### Oxygen consumption rates

Oxygen consumption rates (OCRs) were measured as previously described [24] with minor modification using Seahorse Bioscience XFp Analyzer (Agilent, USA). For the measurement of OCRs, cells were incubated in XF DMEM base medium (pH 7.4; Agilent, USA) containing 25 mM D-glucose (Sigma, USA) and 4 mM L-glutamine (Sigma, USA) at 37 °C. XFp Cell Mito Stress Test Kit (Agilent, USA) was used (2.5 μM of oligomycin, 0.5 μM of FCCP and 0.5 μM of rotenone/antimycin A) [21]. Basal and maximal respiration was calculated by subtraction of non-mitochondrial respiration. Proton leak related OCR was calculated by subtracting rotenone/antimycin A induced OCR from the oligomycin A-induced OCR [24]. OCRs were normalized with protein concentrations. The data were analyzed by Agilent Wave software (version 2.6.0.31).

### Bioluminescence imaging

In vivo bioluminescence imaging was performed as described previously [19]. Briefly, the region of interest of CIDEA reporter mice was shaved with an electric razor. D-luciferin (150 mg/kg, i.p., Goldbio, USA) were injected to CIDEA reporter mice and anesthetized with isoflurane gas (5% isoflurane gas and 2% isoflurane gas was used for induction and maintenance respectively). 10 min after the injection, bioluminescence signals were detected with Ami-X imaging systems (Spectral Instruments Imaging, USA). Images were acquired and quantified with Aura software (version 2.2.1.1).

### Ex vivo triphenyl tetrazolium chloride assay

Ex vivo 2,3,5-triphenyl tetrazolium chloride (TTC, Sigma, USA) staining was performed to assay mitochondrial electron transport activity of adipose tissues [25]. Briefly, each tissue was minced to approximately 30 mg and incubated in PBS with 2% TTC for 15 min at 37 °C. Sequentially, tissues were fixed with 10% formalin for 30 min at room temperature. For the formazan product extraction, each tissue samples were separately transferred into 95% ethanol and incubated at 4 °C overnight. The absorbance of extracted solutions was measured using MultiSkan Go spectrophotometry (Thermo Fisher, USA) at 485 nm and the values were normalized with tissue weights.

### Indirect calorimetry and body composition

After the mice were acclimatized to the system for 2 days, VO_2,_ VCO_2_, energy expenditure (EE), food intake and activity were monitored by PhenoMaster (TSE system, Germany) for the next 3 days. During measurement, mice were kept under condition of 24 °C and 12-h light/12-h dark cycle with food and water provided ad libitum [26]. Fat mass and lean mass of the mice were measured using nuclear magnetic resonance (NMR) scanning EchoMRI-700 (Echo Medical System, India) [26]. Measurement was conducted for 3 min per mouse to minimize the stress of the animals, and no anesthesia was performed.

### Histology and immunohistochemistry

Hematoxylin and eosin (H&E) staining was performed using paraffin sections as previously described [21]. Briefly, adipose tissues were fixed using 10% formalin (Sigma, USA) for 24 h and subjected to paraffin embedding. Subsequently, tissue blocks embedded in paraffin were sectioned into 5 μm thick slice. After deparaffinization, sections were stained using ClearView H&E Y alcoholic solution (BBC biochemical, USA). For immunostaining, deparaffinized sections of adipose tissue were incubated with anti-UCP1 antibody (1:400; UCP11-A, Alpha Diagnostic International, USA) at 4 °C overnight and then with goat anti-rabbit Alexa Flour 488 (1:500; Thermo Fisher, USA) as a secondary antibody for 1 h at room temperature. Nuclei were stained with DAPI (Sigma, USA). LSM800 confocal microscope (Zeiss, Germany) was used to acquire images and the data were analyzed with Zen software (version 3.0).

### Glucose tolerance test

Glucose tolerance tests were performed [27] with 16-week-old mice. Mice were fasted for 12 h before the test, without limiting access to water, and then intraperitoneally injected with 20% D-glucose (2 g/kg, Sigma, USA). Glucose concentrations in blood were measured at indicated time using Gluco Doctor Top meter and strip (Allmedicus, South Korea).

### Fecal lipid extraction

The modified Folch’s extraction method was used for fecal lipid extraction as previously described [28]. Feces of mice were collected for 3 days and fecal lipids were extracted from 1 g of feces per mouse using chloroform:methanol solution (2:1) [28]. The solution was air-dried for 3 days.

### Statistical analysis

Statistical analyses were performed using Prism 7 (version 7.00). Data are presented as mean ± standard errors of the means (SEMs). Unpaired t-test was used to determine statistical significance between 2 groups.

## Results

### HTGT upregulated brown adipocyte marker expression and mitochondrial content in adipocytes in vitro

**Fig. 1:**
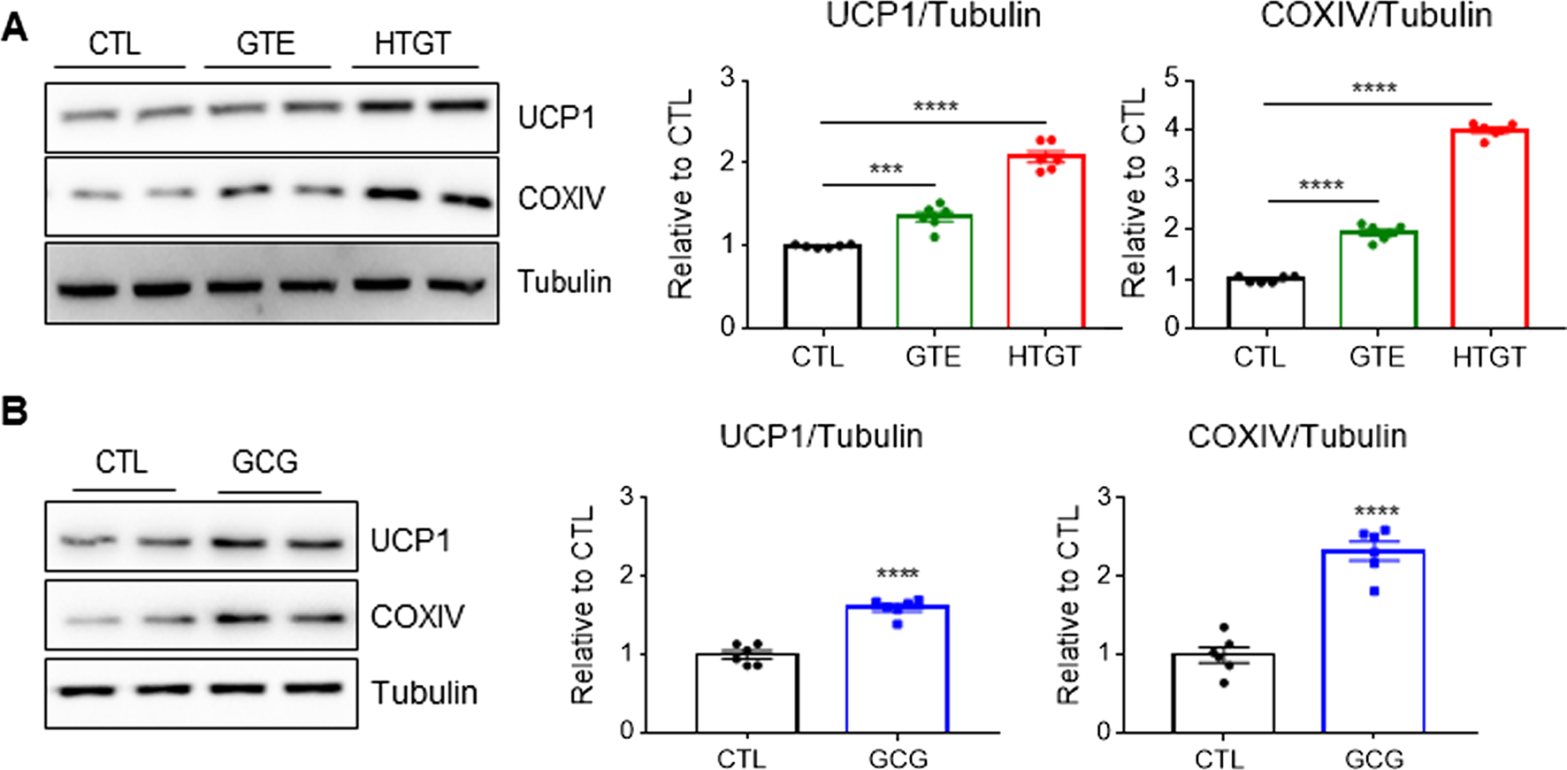
HTGT upregulated mitochondrial content in brown adipocytes. **A** Immunoblot analysis of adipocytes differentiated from immortalized brown preadipocytes treated with vehicle (CTL), GTE (100 μg/mL), or HTGT (100 μg/mL) for 48 h (n = 6). **B** Immunoblot analysis of adipocytes differentiated from immortalized brown preadipocytes treated with vehicle (CTL) or GCG (10 μM) for 48 h (n = 6)

Immortalized brown preadipocytes were differentiated into adipocytes and then treated with GTE or HTGT. Immunoblot analysis was performed to examine the expression levels of the brown adipocyte marker, UCP1, and mitochondrial protein, cytochrome c oxidase subunit 4 (COXIV). GTE increased the protein levels of UCP1 by 1.94 ± 0.07-fold and COXIV by 1.35 ± 0.06-fold (Fig. 1A), while HTGT increased UCP1 by 2.08 ± 0.07-fold and COXIV by 4.0 ± 0.06-fold. The effects of HTGT were significantly greater than those of GTE (Fig. 1A), and the GCG levels in HTGT were higher than those in GTE. Therefore, we hypothesized that GCG is one of the bioactive components responsible for the higher thermogenic activity of HTGT. Indeed, GCG treatment significantly increased UCP1 and COXIV expression in brown adipocytes by 1.6 ± 0.05 -fold and 2.3 ± 0.12-fold, respectively (Fig. 1B).

### Co-treatment with HTGT and EMIQ increased mitochondrial content and metabolism in adipocytes

**Fig. 2:**
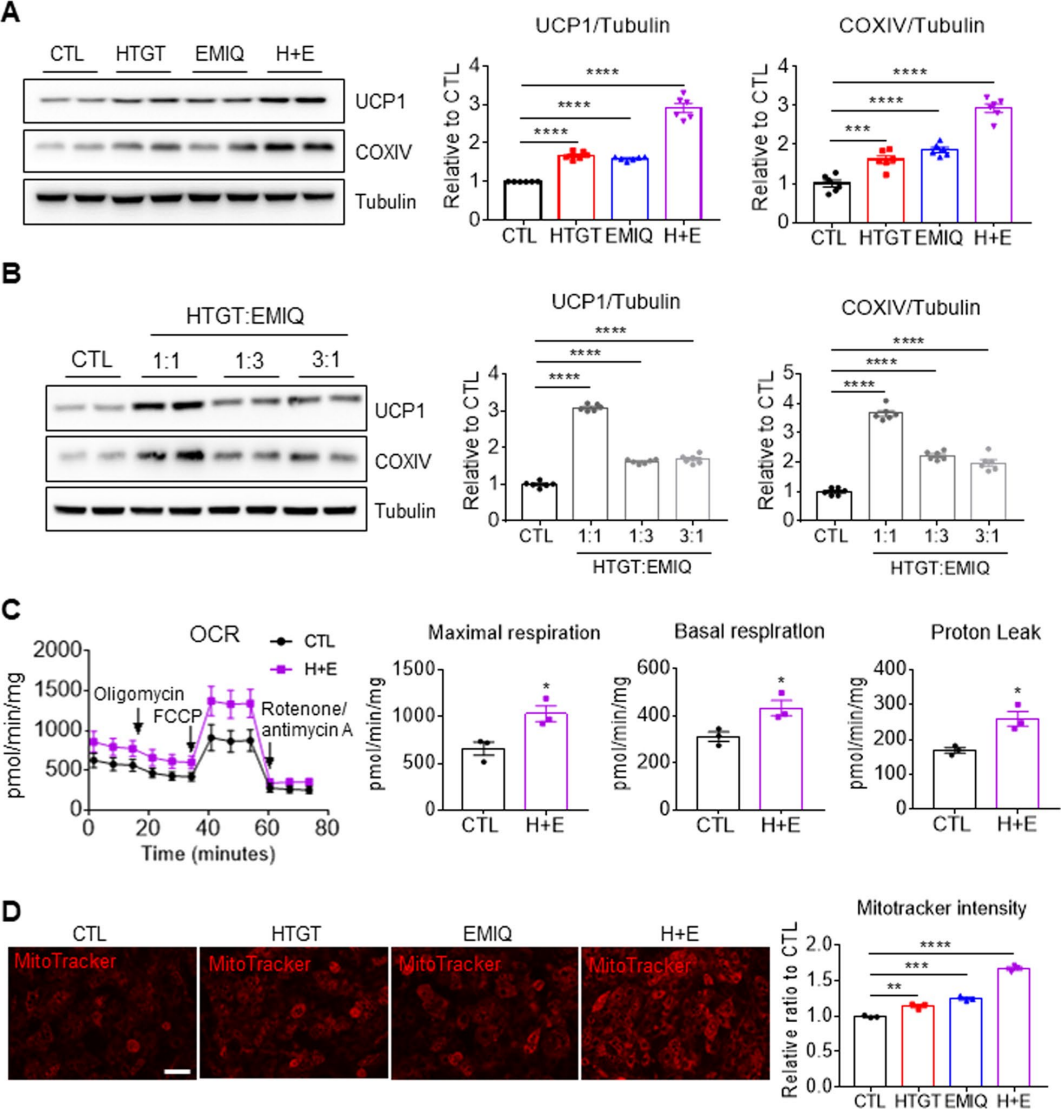
Co-treatment with HTGT and EMIQ synergistically increased mitochondrial content and activity in adipocytes in vitro. **A** Immunoblot analysis of adipocytes differentiated from immortalized brown preadipocytes treated with vehicle control (CTL), HTGT (100 μg/mL), EMIQ (100 μg/mL), or combination of HTGT and EMIQ (H + E, 50 μg/mL + 50 μg/mL) for 48 h (n = 6). **B** Immunoblot analysis of adipocytes differentiated from immortalized brown preadipocytes treated with vehicle control (CTL) or different ratio of combination of HTGT and EMIQ (1:1 (50 μg/mL + 50 μg/mL), 1:3 (25 μg/mL + 75 μg/mL), 3:1 (75 μg/mL + 25 μg/mL)) for 48 h (n = 6). **C** Oxygen consumption rate (OCR) analysis of adipocytes differentiated from immortalized brown preadipocytes treated with vehicle control (CTL) or combination of HTGT and EMIQ (H + E, 50 μg/mL + 50 μg/mL) for 48 h (n = 3). **D** MitoTracker staining of adipocytes differentiated from C3H10T1/2 cells treated with vehicle control (CTL) or HTGT (100 μg/mL), EMIQ (100 μg/mL), combination of HTGT and EMIQ (H + E, 50 μg/mL + 50 μg/mL) for 48 h (Size bar = 50 μm) (n = 3)

Next, the synergistic effects of HTGT and EMIQ, a known thermogenic inducer were investigated [29, 30]. The synergistic effects of co-treatment on brown adipocyte marker expression and mitochondrial content were determined by immunoblot analyses. Single treatment with just HTGT (100 μg/mL) or EMIQ (100 μg/mL) upregulated UCP1 (Fig. 2A. 1.68 ± 0.04-fold-increase by HTGT; 1.60 ± 0.02 fold-increase by EMIQ) and COXIV (1.61 ± 0.097-fold-increase by HTGT; 1.86 ± 0.06-fold-increase by EMIQ). The combination of HTGT and EMIQ had additive effects in brown adipocytes (2.91 ± 0.12 fold-increase in UCP1, 2.93 ± 0.11-fold-increase in COXIV) (Fig. 2A). Various ratios were tested to find the most effective ratio of the mixture, and found that a 1:1 ratio of the combination exhibited the greatest upregulation of UCP1 and COXIV expression (Fig. 2B: (1:1) 3.10 ± 0.04 fold-increase in UCP1, 3.66 ± 0.09 fold-increase in COXIV; (1:3) 1.62 ± 0.02 fold-increase in UCP1, 2.22 ± 0.06 fold-increase in COXIV; (3:1) 1.69 ± 0.05 fold-increase in UCP1, 1.97 ± 0.11 fold-increase in COXIV). Based on the western blot results, the combination of HTGT and EMIQ in a 1:1 ratio was used in all subsequent experiments. Next, effects of HTGT and EMIQ co-treatment on mitochondrial oxygen consumption rates were examined. This functional analysis indicated that co-treatment increased mitochondrial metabolism, including basal, maximal and proton leak-related respiration (Fig. 2C). Moreover, co-treatment with HTGT and EMIQ (HTGT 50 μg/mL, EMIQ 50 μg/mL) increased mitochondrial membrane potential in white adipocytes differentiated from C3H10T1/2 cells, as demonstrated by MitoTracker staining (Fig. 2D).

### Co-treatment with HTGT and EMIQ reduced lipid content in adipocytes

**Fig. 3:**
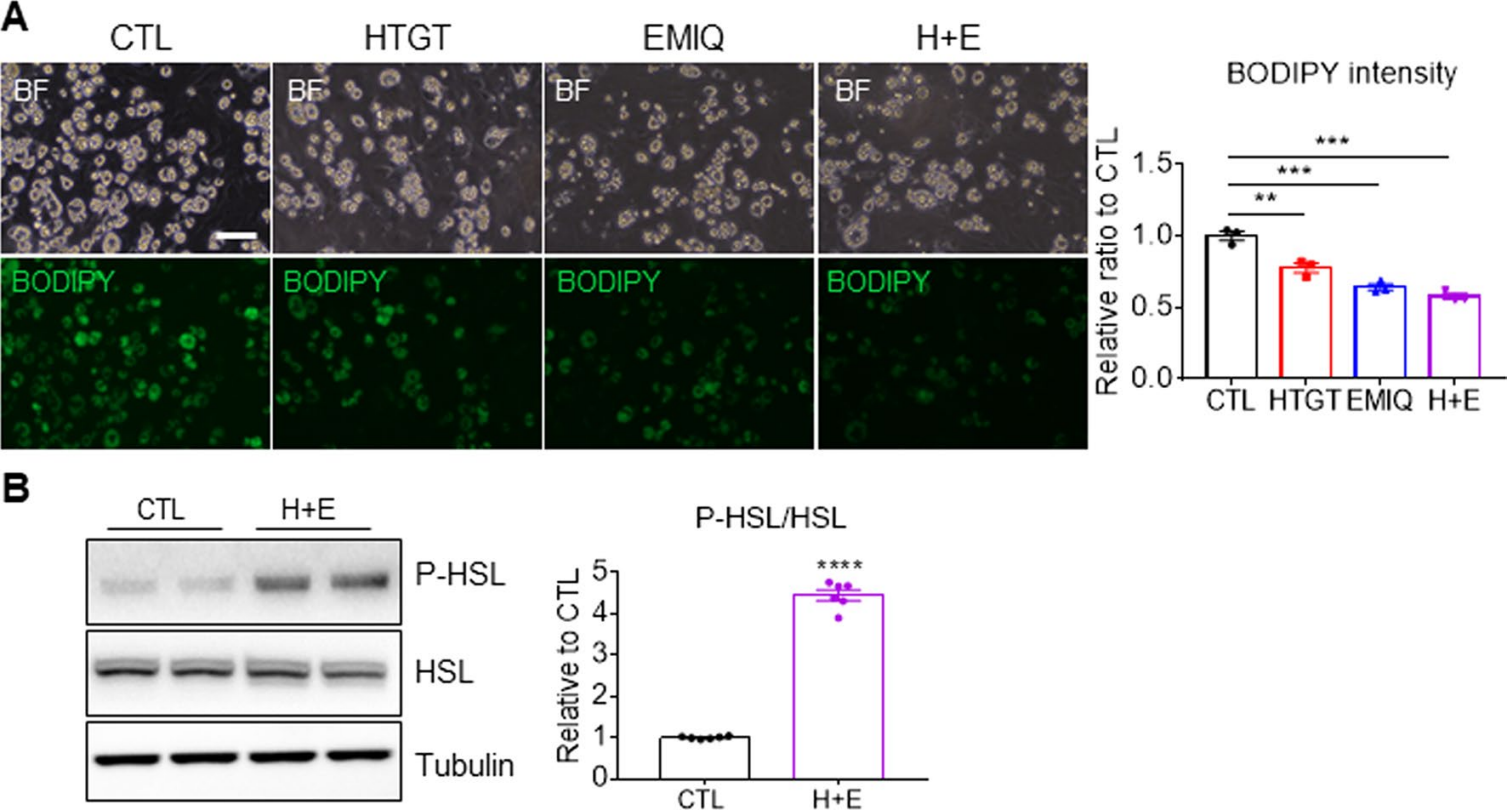
Co-treatment with HTGT and EMIQ increased lipolysis in adipocytes in vitro*.* **A** Representative images and quantification of boron dipyrromethene fluorophore (BODIPY) staining of adipocytes differentiated from immortalized brown preadipocytes treated with vehicle control (CTL), HTGT (100 μg/mL), EMIQ (100 μg/mL), combination of HTGT and EMIQ (H + E, 50 μg/mL + 50 μg/mL) for 24 h (Size bar = 50 μm) (n = 3). **B** Immunoblot analysis of adipocytes differentiated from immortalized brown preadipocytes treated with vehicle control (CTL) or combination of HTGT and EMIQ (H + E, 50 μg/mL + 50 μg/mL) for 24 h (n = 6)

Differentiated adipocytes from immortalized brown preadipocytes were treated with HTGT, EMIQ, or a combination of the two reagents. BODIPY was used to stain neutral lipid, mainly triglyceride in adipocytes. BODIPY staining indicated that single and combination treatment with HTGT and EMIQ reduced lipid content in adipocytes (Fig. 3A). As lipolysis is a major mechanism for reducing lipid content in adipocytes, HSL, a lipase involved in cytosolic lipolysis, was examined. In addition to the expression levels, phosphorylation of HSL at serine 660 was evaluated as an indication of HSL activation. Although HTGT and EMIQ did not affect the expression levels of HSL, the treatment increased the levels of phosphorylated HSL (serine 660) in adipocytes by 4.45 ± 0.13-fold compared to CTL groups (Fig. 3B), indicating its lipolytic effects.

### Co-treatment with HTGT and EMIQ increased brown adipocyte marker expression in adipose tissue in vivo

**Fig. 4:**
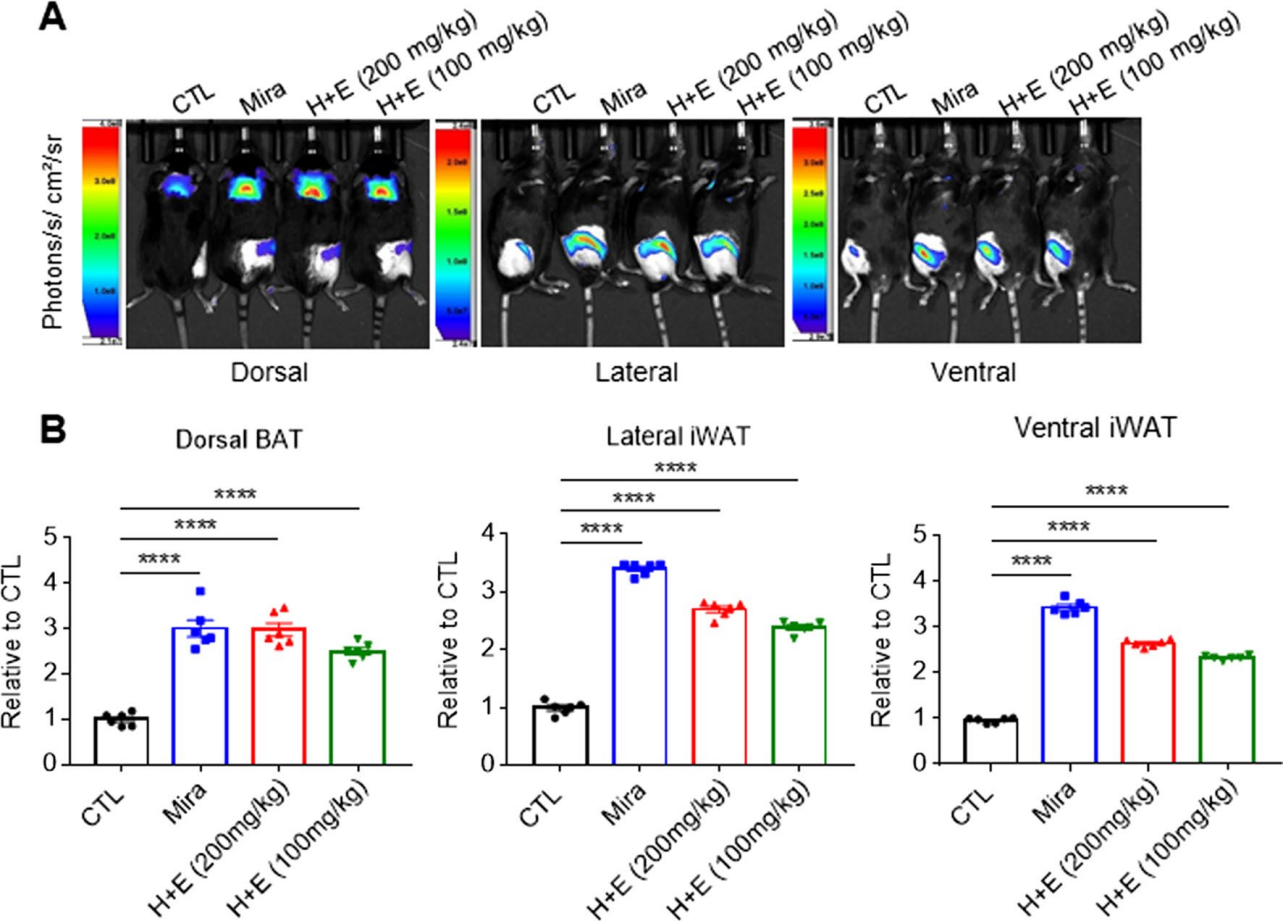
Co-treatment with HTGT and EMIQ induced browning of adipose tissue in vivo*.* **A** In vivo bioluminescence imaging of CIDEA reporter mouse treated with vehicle control (CTL), mirabegron (Mira, 10 mg/kg), or combination of HTGT and EMIQ (H + E, 200 mg/kg (100 mg/kg + 100 mg/kg) or 100 mg/kg (50 mg/kg + 50 mg/kg)) for 2 weeks (n = 6). **B** Quantification of bioluminescence in dorsal brown adipose tissue (BAT), lateral inguinal white adipose tissue (iWAT) and ventral iWAT from (**A**) (n = 6)

**Fig. 5:**
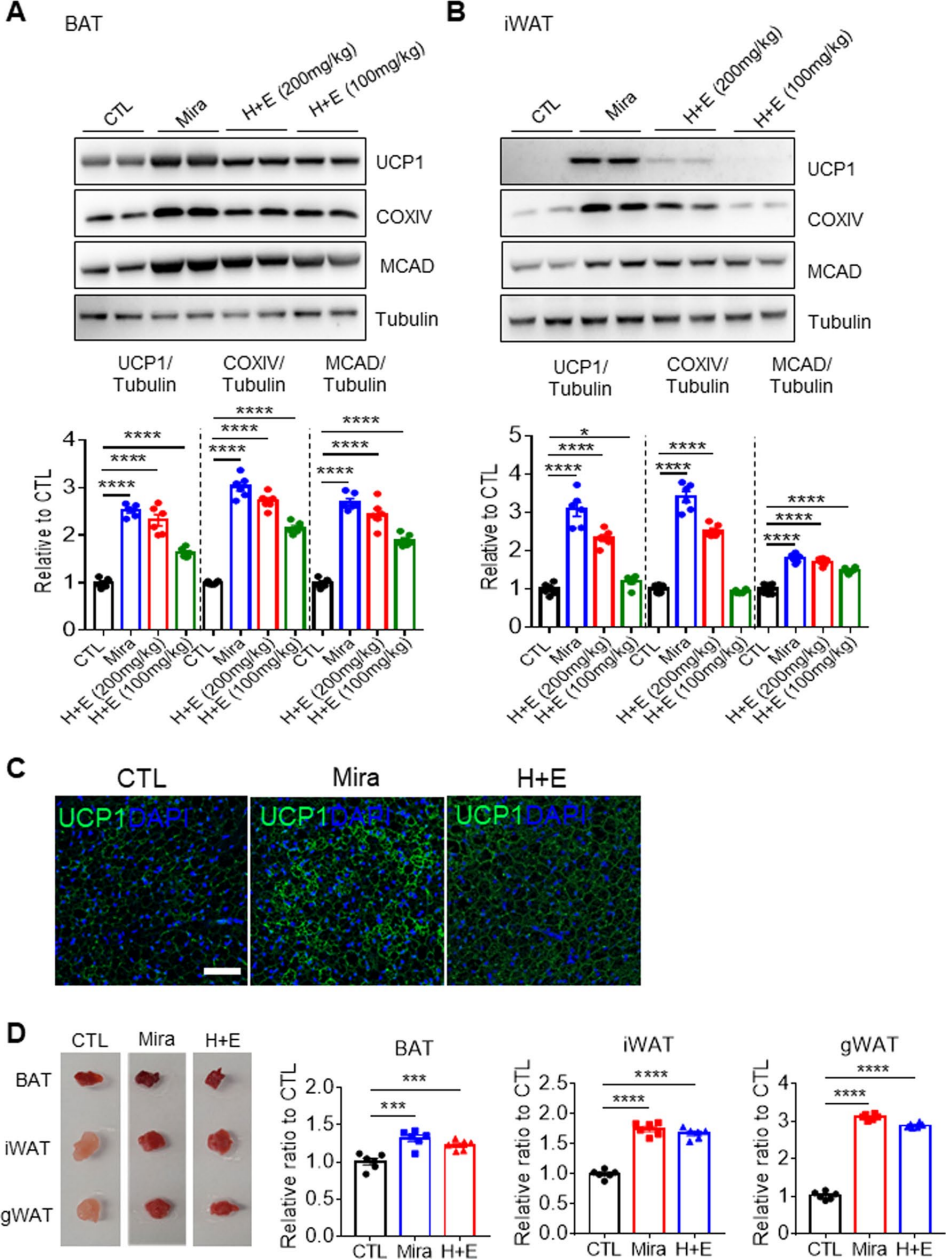
Co-treatment with HTGT and EMIQ upregulated mitochondrial content and activity in adipose tissue in vivo*.* **A**, **B** Immunoblot analysis of brown adipose tissue (BAT) and inguinal white adipose tissue (iWAT) treated with vehicle (CTL), mirabegron (Mira, 10 mg/kg), or combination of HTGT and EMIQ (H + E, 200 mg/kg, 100 mg/kg each) or 100 mg/kg (50 mg/kg each)) for 2 weeks (n = 6). **C** Representative images UCP1 immunostaining of paraffin sections from iWAT of mice treated with vehicle (CTL), mirabegron (Mira, 10 mg/kg) or combination of HTGT and EMIQ (H + E, 100 mg/kg each) for 2 weeks (Size bar = 50 μm). **D** Triphenyltetrazolium chloride staining of BAT, iWAT and gonadal white adipose tissue (gWAT) from mice treated with vehicle (CTL), mirabegron (Mira, 10 mg/kg) or combination of HTGT and EMIQ (H + E, 200 mg/kg (100 mg/kg each) for 2 weeks (n = 6)

Next, in vivo effects of co-treatment with HTGT and EMIQ on adipose tissue browning were examined. CIDEA reporter mice were used to determine the most effective treatment dosage. CIDEA reporter mice were previously developed to monitor CIDEA expression through fluorescence and luminescence signals [19]. The effects of high-dose (HTGT + EMIQ: 100 mg/kg each) and low-dose (HTGT + EMIQ: 50 mg/kg each) administration were compared, and mirabegron (10 mg/kg), a well-known beta-3 adrenergic receptor (β3-AR) agonist, was used as a positive control. Two weeks of treatment with a combination of HTGT and EMIQ increased the bioluminescence intensities of BAT and iWAT. The increase in the high-dose treated group was greater than in the low-dose group; particularly, the levels of BAT in the high-dose group showed an increase comparable with that of the mirabegron-treated group (Fig. 4A, B). In particular, high and low doses of co-treatment increased the bioluminescence intensity of BAT by 2.98 ± 0.14- and 2.51 ± 0.08-fold, respectively (Fig. 4B). In lateral and ventral iWAT, there were 2.7 ± 0.07- and 2.65 ± 0.03-fold increase by high dose, while 2.39 ± 0.06- and 2.34 ± 0.02-fold increase by low dose, respectively (Fig. 4B). Consistent with in vivo imaging results, immunoblot analysis indicated a greater effect of high-dose treatment on browning and mitochondrial contents compared to low doses (Fig. 5A, B). Therefore, we used a combination of HTGT and EMIQ at a high dose in subsequent experiments.

The induction of UCP1 expression in BAT by the high dose co-treatment was further validated by immunohistochemical analysis (Fig. 5C). Additionally, TTC staining indicated upregulation of mitochondrial electron transport in adipose tissue of mice treated with a combination of HTGT and EMIQ (Fig. 5D: 1.23 ± 0.03 fold-increase in BAT, 1.68 ± 0.04 fold-increase in iWAT, 2.89 ± 0.03 fold-increase in gWAT).

### Anti-obesity effects of HTGT and EMIQ in high-fat-diet induced obesity mouse model

**Fig. 6:**
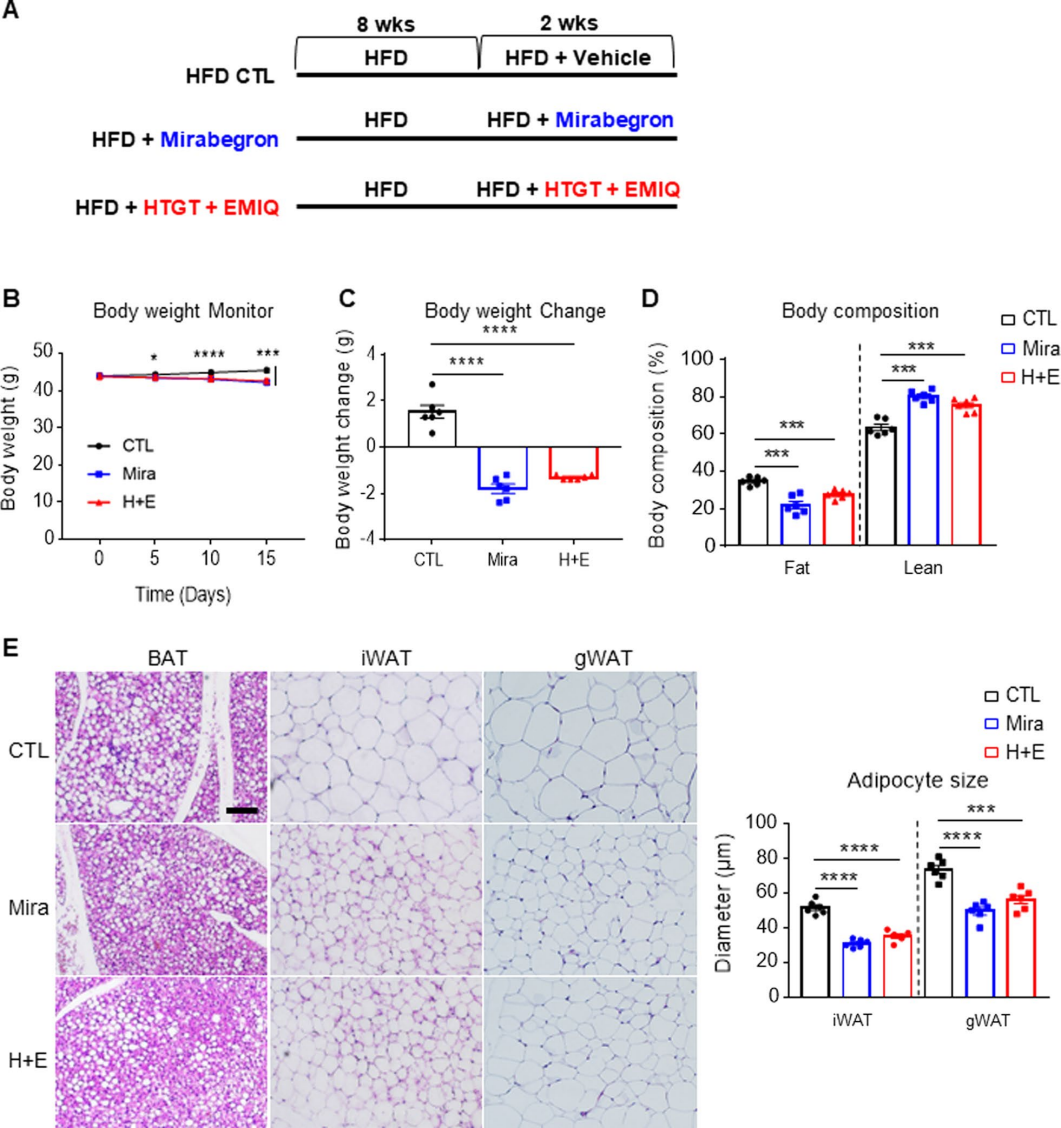
Co-treatment with HTGT and EMIQ protected mice from high-fat diet-induced obesity. **A** Schematic diagram of experimental design and treatment schedule. **B** Body weight monitoring of high-fat diet (HFD) fed mice treated with vehicle control (CTL), mirabegron (Mira, 10 mg/kg) and combination of HTGT and EMIQ (H + E, 100 mg/kg each) (n = 6). **C** Body weight change of HFD fed mice treated with vehicle control (CTL), mirabegron (Mira, 10 mg/kg) and combination of HTGT and EMIQ (H + E, 100 mg/kg each) during 2 weeks of treatment (n = 6). **D** Body composition of fat and lean of HFD fed mice treated with vehicle control (CTL), mirabegron (Mira, 10 mg/kg) and combination of HTGT and EMIQ (H + E, 100 mg/kg each) (n = 6). **E** Hematoxylin and eosin (H&E) staining of brown adipose tissue (BAT), inguinal white adipose tissue (iWAT) and gonadal white adipose tissue (gWAT) obtained from HFD fed mice treated with vehicle control (CTL), mirabegron (Mira, 10 mg/kg) and combination of HTGT and EMIQ (H + E, 100 mg/kg each) (Size bar = 50 μm) (n = 6)

An HFD-induced obesity mouse model was used to assess the anti-obesity effects of HTGT and EMIQ. As shown in Fig. 6A, mice were treated with mirabegron or a combination of HTGT and EMIQ for 2 weeks after 8 weeks of HFD feeding. During the 2 weeks of treatment, mice treated with HTGT and EMIQ exhibited a significant reduction in body weight and fat mass (Fig. 6B–D). Moreover, co-treatment reduced the adipocyte size in all the adipose tissue depots, which was observed using H&E staining (Fig. 6E), indicating protective effects against adipocyte hypertrophy. However, the treatment did not affect fecal fat content, suggesting that HTGT and EMIQ may not affect intestinal lipid absorption (Additional file 1: Fig. S1).

**Fig. 7:**
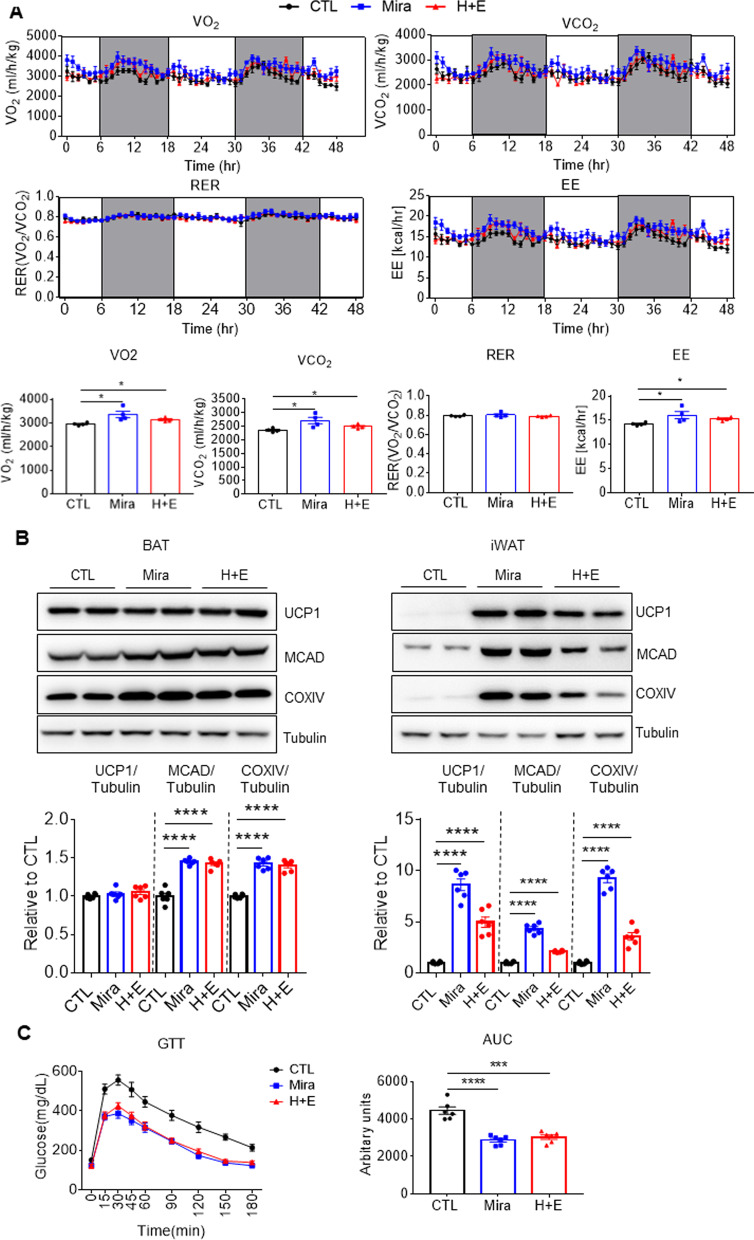
Co-treatment with HTGT and EMIQ increased energy expenditure and improved obesity-induced metabolic dysfunction. **A** Indirect calorimetry analysis of high-fat fed (HFD) mice treated with vehicle control (CTL), mirabegron (Mira, 10 mg/kg) and combination of HTGT and EMIQ (H + E, 100 mg/kg each) (n = 4). **B** Immunoblot analysis of brown adipose tissue (BAT) and inguinal white adipose tissue (iWAT) obtained from HFD fed mice treated with vehicle control (CTL), mirabegron (Mira, 10 mg/kg) and combination of HTGT and EMIQ (H + E, 100 mg/kg each) (n = 6). **C** Intraperitoneal glucose tolerance test (GTT) of HFD fed mice treated with vehicle control (CTL), mirabegron (Mira, 10 mg/kg) and combination of HTGT and EMIQ (H + E, 100 mg/kg each) (n = 6)

Furthermore, mice treated with HTGT and EMIQ exhibited greater VO_2,_ VCO_2_ and energy expenditure (EE) (Fig. 7A). Combination treatment also elevated levels of brown adipocyte markers and mitochondrial proteins, including UCP1, COXIV and medium-chain acyl-CoA dehydrogenase (MCAD) in BAT and iWAT (Fig. 7B BAT: 1.42 ± 0.02 fold-increase in MCAD, 1.40 ± 0.03 fold-increase in COXIV; iWAT: 4.96 ± 0.51 fold-increase in UCP1, 2.09 ± 0.04 fold-increase in MCAD, 3.56 ± 0.37 fold-increase in COXIV). In addition, improvements in glucose tolerance were observed in mice treated with HTGT and EMIQ, whose effect was comparable to that of mirabegron (Fig. 7C).

Collectively, these results indicate the therapeutic potential of HTGT and EMIQ in obesity-related diseases.

## Discussion

Activation of adipose tissue thermogenesis has gained significant attention as a therapeutic target for obesity and related metabolic diseases [5, 26]. Therefore, great efforts have been made to develop reagents that can activate BAT and recruit brown adipocytes in WAT for anti-obesity therapeutics. In this regard, we aimed to determine the potent browning inducers among the phytochemicals tested in this study. We demonstrated that a 1:1 combination of HTGT and EMIQ potently increased brown adipocyte marker expression and mitochondrial content in adipocytes differentiated from immortalized brown pre-adipocytes, indicating that HTGT and EMIQ activate thermogenic metabolism of brown adipocytes. Moreover, in vivo co-treatment with HTGT and EMIQ reduced body weight, increased energy expenditure, and improved glucose tolerance in a diet-induced obesity model.

The current study demonstrated that HTGT had a significantly greater effect on the induction of UCP1 expression than GTE. While the thermogenic activity of EGCG in human and rodent models is controversial [18, 31], our previous studies reported that EGCG did not significantly affect UCP1 expression in white adipocytes [18]. As the heating process can increase GCG levels and reduce content in GTE, we speculated that GCG might be one of the bioactive constituents responsible for thermogenic activity. Indeed, GCG increased UCP1 expression in adipocytes in vitro. Further investigation of the in vivo thermogenic effects of GCG and other constituents of HTGT is required to characterize the molecular mechanisms underlying the thermogenic activity of HTGT.

In the present study, we demonstrated that the combination of HTGT and EMIQ exerts anti-obesity effects, comparable to those of mirabegron, a clinically available β3-AR agonist [32]. The β3-AR signaling pathway is one of the primary regulatory mechanisms involved in thermogenesis in adipose tissue. Its agonists have been investigated as potential anti-obesity drugs [29, 33–36]. Consequently, although mirabegron is commonly used to treat overactive bladder syndrome [32], clinical trials to evaluate its anti-obesity effects have been conducted recently [37]. However, it has also been suggested that mirabegron can accelerate atherosclerotic plaque development through UCP1-dependent lipolysis [38], suggesting potential adverse effects in metabolic disorders.

We investigated the effects of a combination of HTGT and EMIQ on the browning of WAT. Previous studies indicated that EMIQ downregulates adipocyte differentiation and, simultaneously upregulates fatty acid oxidation and WAT browning through the activation of AMP-activated protein kinase (AMPK) α in vivo [30]. Another study suggested that quercetin increases WAT browning and BAT nonshivering thermogenesis by upregulating β3-AR, mitogen-activated protein kinase (MAPK), and AMPK pathways in an HFD-induced obesity model in mouse [39]. Further investigation is required to understand the molecular mechanisms underlying the anti-obesity effects of HTGT and EMIQ.

The in vivo browning effect was monitored using non-invasive imaging of a CIDEA reporter mouse model. Noninvasive molecular imaging approaches enable a more efficient dose–response assessment than conventional drug screening methods that require invasive tissue sampling. In this study, 2 weeks of high-dose treatment demonstrated greater induction of CIDEA expression in BAT and iWAT than low-dose treatment. Thus, the high dose was selected as the optimal dose to test in vivo anti-obesity effects in an HFD-induced obese mouse model. Although not investigated in this study, low-dose, long-term effects warrant further investigation as they are more relevant to the clinical application than short-term effects, considering the general consumption patterns of dietary supplements [40].

In this study, we focused on the effects of HTGT and EMIQ on adipose tissue metabolism. Although adipose tissue is a central metabolic organ involved in the pathogenesis of obesity-related metabolic diseases, the potential health benefits of HTGT and EMIQ treatment, such as insulin sensitivity and anti-inflammatory effects in other metabolic organs, need further investigation.

Combination treatment can amplify the effects of reagents synergistically as multiple signaling pathways can be targeted simultaneously. Several non-canonical pathways for the induction of thermogenic programs have been identified; therefore, a combination treatment targeting these multiple molecular players in thermogenic programs would be a powerful strategy to combat obesity-related metabolic disorders. Metabolic syndrome is also associated with multiple pathologic events and conditions in various metabolic organs; thus, combination drug therapies would have greater efficacy than single medication therapies.

## Conclusions

In summary, we investigated the synergistic anti-obesity effects of a combination of HTGT and EMIQ using in vitro adipocytes and an HFD-induced obesity mouse model. Our findings suggest that a combination of HTGT and EMIQ is a promising therapeutic agent for the treatment of obesity and related metabolic diseases.

## Supplementary Information

**Additional file 1: Table S1.** Antibodies used for immunoblot analysis. **Figure S1.** Co-treatment of HTGT and EMIQ dose not affect fecal lipid contents.

## Data Availability

Data are all contained within the article.

## Abbreviations

HTGT: Heat-transformed green tea
EMIQ: Enzymatically modified isoquercitrin
BAT: Brown adipose tissue
WAT: White adipose tissue
PKA: CAMP-dependent protein kinase A
HSL: Hormone-sensitive lipase
ATGL: Adipose triglyceride lipase
UCP1: Uncoupling protein 1
GTE: Green tea extract
EGCG: Epigallocatechin-3-gallate
β3-AR: β3-adrenergic receptor
MAPK: Mitogen-activated protein kinase
AMPK: AMP-activated protein kinase
HFD: High-fat diet
CIDEA: DNA fragmentation factor-like effector A
COXIV: Cytochrome c oxidase subunit 4
GCG: Gallocatechin-3-gallate
MCAD: Medium-chain acyl-CoA dehydrogenase

## References

[1] Hill JO, Melanson EL (1999). Overview of the determinants of overweight and obesity: current evidence and research issues. Med Sci Sports Exerc.

[2] Andolfi C, Fisichella PM (2018). Epidemiology of obesity and associated comorbidities. J Laparoendosc Adv Surg Tech A.

[3] Fenzl A, Kiefer FW (2014). Brown adipose tissue and thermogenesis. Horm Mol Biol Clin Investig.

[4] Duncan RE, Ahmadian M, Jaworski K, Sarkadi-Nagy E, Sul HS (2007). Regulation of lipolysis in adipocytes. Annu Rev Nutr.

[5] Lee YH, Mottillo EP, Granneman JG (2014). Adipose tissue plasticity from WAT to BAT and in between. Biochim Biophys Acta.

[6] Kohara A, Machida M, Setoguchi Y, Ito R, Sugitani M, Maruki-Uchida H, Inagaki H, Ito T, Omi N, Takemasa T (2017). Enzymatically modified isoquercitrin supplementation intensifies plantaris muscle fiber hypertrophy in functionally overloaded mice. J Int Soc Sports Nutr.

[7] Li F, Gao C, Yan P, Zhang M, Wang Y, Hu Y, Wu X, Wang X, Sheng J (2018). EGCG reduces obesity and white adipose tissue gain partly through AMPK activation in mice. Front Pharmacol.

[8] Remely M, Ferk F, Sterneder S, Setayesh T, Roth S, Kepcija T, Noorizadeh R, Rebhan I, Greunz M, Beckmann J (2017). EGCG prevents high fat diet-induced changes in gut microbiota, decreases of DNA strand breaks, and changes in expression and DNA methylation of Dnmt1 and MLH1 in C57BL/6J male mice. Oxid Med Cell Longev.

[9] Zhou J, Mao L, Xu P, Wang Y (2018). Effects of (-)-epigallocatechin gallate (EGCG) on energy expenditure and microglia-mediated hypothalamic inflammation in mice fed a high-fat diet. Nutrients.

[10] Chen IJ, Liu CY, Chiu JP, Hsu CH (2016). Therapeutic effect of high-dose green tea extract on weight reduction: a randomized, double-blind, placebo-controlled clinical trial. Clin Nutr.

[11] Basu A, Sanchez K, Leyva MJ, Wu M, Betts NM, Aston CE, Lyons TJ (2010). Green tea supplementation affects body weight, lipids, and lipid peroxidation in obese subjects with metabolic syndrome. J Am Coll Nutr.

[12] Nagao T, Hase T, Tokimitsu I (2007). A green tea extract high in catechins reduces body fat and cardiovascular risks in humans. Obesity (Silver Spring).

[13] Kim ES, Liang YR, Jin J, Sun QF, Lu JL, Du YY, Lin C (2007). Impact of heating on chemical compositions of green tea liquor. Food Chem.

[14] Wang L-F, Kim D-M, Lee CY (2000). Effects of heat processing and storage on flavanols and sensory qualities of green tea beverage. J Agric Food Chem.

[15] Ikeda I, Hamamoto R, Uzu K, Imaizumi K, Nagao K, Yanagita T, Suzuki Y, Kobayashi M, Kakuda T (2005). Dietary gallate esters of tea catechins reduce deposition of visceral fat, hepatic triacylglycerol, and activities of hepatic enzymes related to fatty acid synthesis in rats. Biosci Biotechnol Biochem.

[16] Bae HJ, Kim J, Jeon SJ, Kim J, Goo N, Jeong Y, Cho K, Cai M, Jung SY, Kwon KJ, Ryu JH (2020). Green tea extract containing enhanced levels of epimerized catechins attenuates scopolamine-induced memory impairment in mice. J Ethnopharmacol.

[17] Hasumura M, Yasuhara K, Tamura T, Imai T, Mitsumori K, Hirose M (2004). Evaluation of the toxicity of enzymatically decomposed rutin with 13-weeks dietary administration to Wistar rats. Food Chem Toxicol.

[18] Kim SN, Kwon HJ, Akindehin S, Jeong HW, Lee YH (2017). Effects of epigallocatechin-3-gallate on autophagic lipolysis in adipocytes. Nutrients.

[19] Son Y, Choi C, Song C, Im H, Cho YK, Son JS, Joo S, Joh Y, Lee YJ, Seong JK, Lee Y-H (2021). Development of CIDEA reporter mouse model and its application for screening thermogenic drugs. Sci Rep.

[20] Poekes L, Gillard J, Farrell GC, Horsmans Y, Leclercq IA (2019). Activation of brown adipose tissue enhances the efficacy of caloric restriction for treatment of nonalcoholic steatohepatitis. Lab Invest.

[21] Cho YK, Son Y, Saha A, Kim D, Choi C, Kim M, Park J-H, Im H, Han J, Kim K (2021). STK3/STK4 signalling in adipocytes regulates mitophagy and energy expenditure. Nat Metab.

[22] Liu D, Lin Y, Kang T, Huang B, Xu W, Garcia-Barrio M, Olatinwo M, Matthews R, Chen YE, Thompson WE (2012). Mitochondrial dysfunction and adipogenic reduction by prohibitin silencing in 3T3-L1 cells. PLoS ONE.

[23] Warnke I, Goralczyk R, Fuhrer E, Schwager J (2011). Dietary constituents reduce lipid accumulation in murine C3H10 T1/2 adipocytes: a novel fluorescent method to quantify fat droplets. Nutr Metab.

[24] Bugge A, Dib L, Collins S, MacDougald OA (2014). Chapter thirteen: measuring respiratory activity of adipocytes and adipose tissues in real time. Methods in enzymology.

[25] Li P, Zhu Z, Lu Y, Granneman JG (2005). Metabolic and cellular plasticity in white adipose tissue II: role of peroxisome proliferator-activated receptor-alpha. Am J Physiol Endocrinol Metab.

[26] Kim SN, Ahn SY, Song HD, Kwon HJ, Saha A, Son Y, Cho YK, Jung YS, Jeong HW, Lee YH (2020). Antiobesity effects of coumestrol through expansion and activation of brown adipose tissue metabolism. J Nutr Biochem.

[27] Ayala JE, Samuel VT, Morton GJ, Obici S, Croniger CM, Shulman GI, Wasserman DH, McGuinness OP (2010). Standard operating procedures for describing and performing metabolic tests of glucose homeostasis in mice. Dis Model Mech.

[28] Kraus D, Yang Q, Kahn BB (2015). Lipid extraction from mouse feces. Bio Protoc.

[29] Nahon KJ, Janssen LGM, Sardjoe Mishre ASD, Bilsen MP, van der Eijk JA, Botani K, Overduin LA, Ruiz JR, Burakiewicz J, Dzyubachyk O (2020). The effect of mirabegron on energy expenditure and brown adipose tissue in healthy lean South Asian and Europid men. Diabetes Obes Metab.

[30] Jiang H, Yoshioka Y, Yuan S, Horiuchi Y, Yamashita Y, Croft KD, Ashida H (2019). Enzymatically modified isoquercitrin promotes energy metabolism through activating AMPKα in male C57BL/6 mice. Food Funct.

[31] Choi C, Song HD, Son Y, Cho YK, Ahn SY, Jung YS, Yoon YC, Kwon SW, Lee YH (2020). Epigallocatechin-3-gallate reduces visceral adiposity partly through the regulation of beclin1-dependent autophagy in white adipose tissues. Nutrients.

[32] Takasu T, Ukai M, Sato S, Matsui T, Nagase I, Maruyama T, Sasamata M, Miyata K, Uchida H, Yamaguchi O (2007). Effect of (R)-2-(2-aminothiazol-4-yl)-4'-{2-[(2-hydroxy-2-phenylethyl)amino]ethyl} acetanilide (YM178), a novel selective beta3-adrenoceptor agonist, on bladder function. J Pharmacol Exp Ther.

[33] Arch JR, Ainsworth AT, Cawthorne MA, Piercy V, Sennitt MV, Thody VE, Wilson C, Wilson S (1984). Atypical beta-adrenoceptor on brown adipocytes as target for anti-obesity drugs. Nature.

[34] O'Mara AE, Johnson JW, Linderman JD, Brychta RJ, McGehee S, Fletcher LA, Fink YA, Kapuria D, Cassimatis TM, Kelsey N (2020). Chronic mirabegron treatment increases human brown fat, HDL cholesterol, and insulin sensitivity. J Clin Invest.

[35] Baskin AS, Linderman JD, Brychta RJ, McGehee S, Anflick-Chames E, Cero C, Johnson JW, O'Mara AE, Fletcher LA, Leitner BP (2018). Regulation of human adipose tissue activation, gallbladder size, and bile acid metabolism by a β3-adrenergic receptor agonist. Diabetes.

[36] Hao L, Scott S, Abbasi M, Zu Y, Khan MSH, Yang Y, Wu D, Zhao L, Wang S (2019). Beneficial metabolic effects of mirabegron in vitro and in high-fat diet-induced obese mice. J Pharmacol Exp Ther.

[37] Finlin BS, Memetimin H, Zhu B, Confides AL, Vekaria HJ, El Khouli RH, Johnson ZR, Westgate PM, Chen J, Morris AJ (2020). The β3-adrenergic receptor agonist mirabegron improves glucose homeostasis in obese humans. J Clin Investig.

[38] Sui W, Li H, Yang Y, Jing X, Xue F, Cheng J, Dong M, Zhang M, Pan H, Chen Y (2019). Bladder drug mirabegron exacerbates atherosclerosis through activation of brown fat-mediated lipolysis. Proc Natl Acad Sci.

[39] Pei Y, Otieno D, Gu I, Lee SO, Parks JS, Schimmel K, Kang HW (2021). Effect of quercetin on nonshivering thermogenesis of brown adipose tissue in high-fat diet-induced obese mice. J Nutr Biochem.

[40] Block G, Jensen CD, Norkus EP, Dalvi TB, Wong LG, McManus JF, Hudes ML (2007). Usage patterns, health, and nutritional status of long-term multiple dietary supplement users: a cross-sectional study. Nutr J.

