# Supplementary material for: Anti-obesity effects of heat-transformed green tea extract through the activation of adipose tissue thermogenesis
Source: Nutr Metab (Lond). 2022 Mar 3;19:14. doi: 10.1186/s12986-022-00648-6 (PMC8896087; doi:10.1186/s12986-022-00648-6)
Supplement: Supplementary file 1 — Additional file 1: Table S1. Antibodies used for immunoblot analysis. Figure S1. Co-treatment of HTGT and EMIQ dose not affect fecal lipid contents. [file 12986_2022_648_MOESM1_ESM.pdf]

Supplementary Table 1. Antibodies used for immunoblot analysis

Antibody	Host	Company	Catalog #	Dilution
HSL	Rabbit	Cell Signaling	4107	1:1000
Phospho-HSL	Rabbit	Cell Signaling	45804	1:1000
UCP1	Rabbit	Alpha Diagnostic International	UCP11-A	1:1000
COX IV	Rabbit	Cell Signaling	4850	1:1000
MCAD	Mouse	Santa Cruz	sc-365030	1:1000
Tubulin	Rabbit	Cell Signaling	2148	1:1000

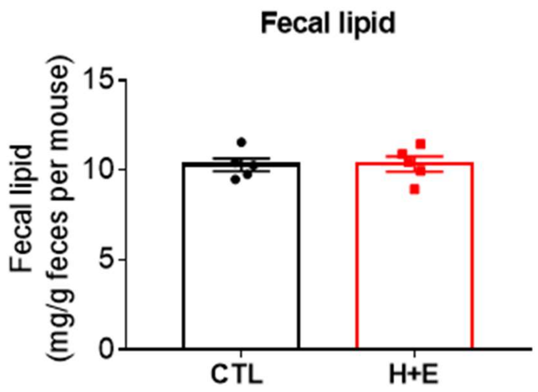

Supplementary Figure 1. Effects of co-treatment of HTGT and EMIQ on fecal lipid content

Lipid content in feces of HFD-fed mice treated with vehicle (CTL) or combination of HTGT and EMIQ (H+E, 100 mg/kg each) (n = 6)
